# The Neuropeptide VIP Limits Human Osteoclastogenesis: Clinical Associations with Bone Metabolism Markers in Patients with Early Arthritis

**DOI:** 10.3390/biomedicines9121880

**Published:** 2021-12-10

**Authors:** David Castro-Vazquez, Amalia Lamana, Paula Arribas-Castaño, Irene Gutiérrez-Cañas, Raúl Villanueva-Romero, Selene Pérez-García, Carmen Martínez, Yasmina Juarranz, Sara Fernández de Córdoba, Isidoro González-Álvaro, Rosa P. Gomariz, Mar Carrión

**Affiliations:** 1 Department of Cell Biology, Faculty of Biology and Faculty of Medicine, Complutense University of Madrid (UCM), 28040 Madrid, Spain; dcastr01@ucm.es (D.C.-V.); amaliala@ucm.es (A.L.); pauarr02@ucm.es (P.A.-C.); irgutier@ucm.es (I.G.-C.); ravillan@ucm.es (R.V.-R.); selene@ucm.es (S.P.-G.); cmmora@ucm.es (C.M.); yashina@ucm.es (Y.J.); macarrio@ucm.es (M.C.); 2 Rheumatology Department, Hospital Universitario La Princesa, Instituto de Investigación Sanitaria Hospital Universitario de La Princesa, 28006 Madrid, Spain; sarfdezco@gmail.com (S.F.d.C.); isidoro.ga@ser.es (I.G.-Á.)

**Keywords:** VIP, arthritis, osteoclastogenesis, bone erosion, osteoclast, αvβ3 integrin, actin cytoskeleton, NFATc1

## Abstract

We aimed to evaluate the direct action of VIP on crucial molecules involved in human osteoclast differentiation and function. We also investigated the relationship between VIP serum levels and bone remodeling mediators in early arthritis patients. The expression of VIP receptors and osteoclast gene markers in monocytes and in vitro differentiated osteoclasts was studied by real-time PCR. NFATc1 activity was measured using a TransAM^®^ kit. Osteoclastogenesis was confirmed by quantification of tartrate-resistant acid phosphatase positive multinucleated cells. OsteoAssay^®^ Surface Multiple Well Plate was used to evaluate bone-resorbing activity. The ring-shaped actin cytoskeleton and the VPAC1 and VPAC2 expression were analyzed by immunofluorescence. We described the presence of VIP receptors in monocytes and mature osteoclasts. Osteoclasts that formed in the presence of VIP showed a decreased expression of osteoclast differentiation gene markers and proteolytic enzymes involved in bone resorption. VIP reduced the resorption activity and decreased both β3 integrin expression and actin ring formation. Elevated serum VIP levels in early arthritis patients were associated with lower BMD loss and higher serum OPG concentration. These results demonstrate that VIP exerts an anti-osteoclastogenic action impairing both differentiation and resorption activity mainly through the negative regulation of NFATc1, evidencing its bone-protective effects in humans.

## 1. Introduction

Bone remodeling maintains skeletal integrity thanks to the coordinated activity of osteoclasts and osteoblasts. Therefore, when the activity of these cells is uncoupled, the physiological balance between bone resorption and formation is altered, with pathological repercussions on bone tissue [1,2]. Such is the case of rheumatoid arthritis (RA), an inflammatory autoimmune disease characterized by a chronic inflammation of the diarthrodial joints and articular bone erosions, mainly attributed to the hyperactivation of osteoclasts [3,4].

Osteoclasts are large multinucleated cells resulting from the fusion and differentiation of mononuclear precursors, found in bone marrow as well as in the monocyte fraction of peripheral blood, through a process known as osteoclastogenesis [5]. The resorptive activity of mature osteoclasts is initiated with their adhesion to bone matrix. This process is mediated by αvβ3 transmembrane integrins and strongly determined by the reorganization of the actin cytoskeleton into a peripheral ring that interacts with integrins to form a sealing zone around the resorption compartment [6,7].

The driving force of osteoclastogenesis is the interaction of the receptor activator of NF-κB ligand (RANKL) with its specific receptor RANK on osteoclast precursors in the presence of macrophage colony-stimulating factor (M-CSF) which expands the osteoclast cellular precursor pool [8,9]. The RANKL signaling pathway finally induces the expression and activation of NFATc1, recognized as the master transcription factor in the regulation of osteoclast differentiation [10]. NFATc1 translocates into the nucleus where it targets numerous osteoclast-specific genes encoding for proteins and enzymes involved in cell differentiation and fusion, such as the dendritic cell-specific transmembrane protein (DC-STAMP), as well as in resorptive function, including tartrate-resistant acid phosphatase (TRAP), integrin β3, cathepsin K (CTSK) and matrix metalloproteinase 9 (MMP9) [11,12,13]. In addition to the RANK/RANKL pair, there is a third molecule considered a key player in the orchestration of osteoclastogenesis. Osteoprotegerin (OPG) is a soluble decoy receptor for RANKL that prevents its interaction with RANK, thereby blocking osteoclast differentiation. Accordingly, the balance between RANKL and OPG is critical in regulating the extent to which osteoclastogenesis occurs [14]. In joints affected by RA, the presence of proinflammatory cytokines and autoantibodies along with an increased RANKL/OPG ratio provides a pro-osteoclastogenic microenvironment that enhances the differentiation of bone-resorbing osteoclasts [15,16,17]. In fact, systemic inflammation in RA patients has been linked to loss of bone mineral density (BMD) and increased risk of fractures [18,19]. In this regard, disease remission has been shown to be associated with maintenance of BMD, whereas active disease is accompanied by higher radiological damage and lower BMD [20,21,22].

A growing body of knowledge demonstrates that bone remodeling is not only modulated by inflammatory mediators and systemic hormones, but also by nervous system in a complex network of neuro-immuno-osteogenic interactions [23,24,25]. In this sense, several neuropeptides detected in the joint microenvironment, including the vasoactive intestinal peptide (VIP), have been proven to elicit modulatory actions on the osteoclastogenic process [26,27,28,29,30]. VIP is a 28 amino acid peptide found in the peripheral and central nervous system that is also expressed by immune and endocrine cells, exerting its biological actions through two specific receptors, VPAC1 and VPAC2 [31,32]. Many studies have demonstrated its potent anti-inflammatory and immunomodulatory properties both in RA animal models and in human ex vivo studies [33]. Moreover, serum VIP levels have been recently identified as a potential prognostic biomarker in early arthritis, as patients with low baseline levels have a poorer clinical course and thus require more intensive treatment [34,35]. VIP treatment has been evidenced to reduce bone erosion in the collagen-induced mouse model of arthritis (CIA) by modulating the synthesis of several osteoclastogenic factors as well as by lowering the RANKL/OPG ratio [36,37]. Furthermore, in vitro studies on rat and mouse osteoclasts have evidenced a direct inhibitory effect of VIP on their differentiation, activation and resorptive function [28,29,30,38]. However, there is still a lack of studies assessing the impact of VIP on the osteoclastogenic potential of human osteoclast progenitors and on their resorptive activity.

In the present study we aimed to examine if the anti-osteoclastogenic effects of VIP described in animal models are also replicated in human cells by analyzing its direct action on crucial molecules and cellular structures involved in human osteoclast differentiation and function. Additionally, we investigated whether there is a relationship between serum levels of VIP and mediators involved in bone remodeling in early arthritis patients. The results of this study will contribute to elucidate the molecular mechanisms likely to be impaired by VIP in human osteoclast biology and to perform a preliminary clinical translational approach to evaluate its potential involvement in modulating bone homeostasis in RA patients.

## 2. Materials and Methods

### 2.1. Study Population

Peripheral blood samples from 12 healthy donors were obtained from the Transfusion Center of the Community of Madrid (CAM, Spain). The study was performed according to the recommendations of the Declaration of Helsinki and was approved by the Ethics Committees of the Transfusion Center of CAM. Following the Spanish Personal Data Protection law, the patients’ demographic information was confidential. All donors signed an informed consent form before sampling.

To address the study of the impact of VIP on bone metabolism in patients with early arthritis, we used the following variables recorded in the Princesa Early Arthritis Register Longitudinal (PEARL) study database: total RANKL serum concentrations, OPG and VIP serum levels and the variation in bone mineral density during the first two years of follow-up. PEARL study includes patients referred to the Early Arthritis Clinic at the Hospital Universitario La Princesa, Madrid, Spain. To be referred to the clinic, patients must have had one or more swollen joints for at least 4 weeks and symptoms for less than a year. Patients with other specific causes of arthritis were excluded. The register’s protocol included five visits during a follow-up period of two years. Baseline blood samples were collected before treatment prescription. At each visit, sociodemographic, clinical and laboratory test data were collected and entered into an electronic database. A more detailed description of the PEARL protocol has been published previously [39].

The Instituto de Investigación Sanitaria La Princesa Research Ethics Committee reviewed and approved the protocol of PEARL study (PI-518), and all experiments were performed in accordance with the guidelines and regulations of this committee. All patients signed an informed consent form before data were included in the register, and biological samples were stored at the local Biobank.

### 2.2. Isolation of PBMCs and Cell Culture

Peripheral blood samples from healthy donors were collected and PBMCs were isolated by Ficoll-Hypaque density gradient (Rafer, Madrid, Spain). For monocyte isolation by adherence, PBMCs were plated at 5 × 10^5^ cells/cm^2^ in 6-, 24- or 48-well plates and allowed to adhere overnight at 37 °C and 5% CO_2_ in α-MEM Glutamax (Gibco, Thermo Fisher Scientific, Madrid, Spain) supplemented with 10% FBS (Gibco, Thermo Fisher Scientific, Madrid, Spain), 1% Penicillin/streptomycin (Sigma-Aldrich, St Louis, MO, USA) and 30 ng/mL M-CSF with or without 10^−8^ M VIP (Bachem A.G., Bubendrof, Switzerland). Non-adherent cells were removed and monocytes were cultured for 14 days in α-MEM Glutamax supplemented with 10% FBS and 1% Penicillin/streptomycin, at 37 °C in a humidified atmosphere containing 10 ng/mL M-CSF, 1 ng/mL RANKL and 5 ng/mL TGFβ-1 (Peprotech, East Windsor, NJ, USA) in the presence or absence of 10^−8^ M VIP.

### 2.3. Quantification of Tartrate-Resistant Acid Phosphatse Positive (TRAP+) Stained Osteoclast

After 14 days of culture, to confirm osteoclastogenesis, cells were fixed and stained using the Leukocyte acid Phosphatase Kit (Sigma-Aldrich) following the manufacturer’s protocol. Assays were made in triplicate; TRAP positive and multinucleated cells (≥3 nuclei) were counted as osteoclasts under a bright field microscope using FIJI-Image J Software 1.52p version.

### 2.4. Resorption Assay

To evaluate bone-resorbing activity in osteoclast-like cells, cells were seeded on OsteoAssay^®^ Surface Multiple Well Plate (Corning, Sigma-Aldrich) under the experimental conditions described above. After 14 days of culture, wells were stained by Von Kossa staining, thus the intact bone matrix was stained in black color whereas the resorption area of osteoclast was visualized in white. Assays were made in triplicate; resorption areas were quantified using FIJI-Image J software 1.52p version.

### 2.5. Real-Time PCR Analysis

Total RNA from cells under osteoclast differentiation conditions for 14 days was extracted using TriReagent method (Sigma-Aldrich, St Louis, MO, USA). RNA quantity and purity were measured on a NanoDrop^®^ and 2 µg was used for cDNA synthesis using a High Capacity cDNA Reverse Transcription Kit (Life Technologies, Carlsbad, CA, USA). Real-time PCR analyses for all target genes (*NFATC1*, *DCSTAMP*, *TNFRSF11A*, *CALCR*, *ACP5*, *CTSK*, *MMP9*, *ITGAV* and *ITGB3*) and one house keeping gene (*GADPH*) were performed using TaqMan Gene Expression Master Mix (Applied Biosystem, Thermo Fisher Scientific), with manufacturer-predesigned primers. VIP receptors (*VIPR1* and *VIPR2*) were tested by semiquantitative RT-PCR using Real-time Ready Assay probes from the Universal Probe Library (Roche Life Science, Barcelona, Spain). Amplification was performed in a LightCycler^®^ 480 Instrument II (Roche Life Science). Assays were made in triplicate, and results were normalized according to the expression levels of *GADPH*.

### 2.6. Transcription Factor Activity Assay (TransAM)

The activity of NFATc1 was assessed on 3 μg nuclear extracts using the TransAM™ NFATc1 Transcription Factor Assay Kit (Active Motif, Carlsbad, USA), according to manufacturer’s instructions. A Nuclear Extract Kit (Active Motif) was used for nuclear extract preparation, and the protein content was measured with a QuantiProTM BCA Assay Kit (Sigma-Aldrich). The results (OD 450/655 nm) were reported as a percentage increase over control cells.

Time-course experiment to evaluate the best time of NFATc1 activation following 1 ng/mL RANKL stimulation was performed, including 6, 12 and 24 h. According to the results obtained, a stimulation time of 12 h was selected.

### 2.7. Inmunofluorescence Microscopy

Visualization of actin ring, VPAC1 and VPAC2 expression was analyzed by immunofluorescence. Cells were seeded on 12 mm coverslips coated with poly-L-Lysine under different experimental conditions and after 14 days were fixed and permeabilized. After rehydration and blocking, actin was stained with 5 U/mL Atto 645N-Phalloidin. For VPAC1 and VPAC2 detection coverslips were incubated with rabbit polyclonal antihuman VPAC1 antibody (1:100, Thermo Fisher Scientific, Madrid, Spain) and mouse monoclonal antihuman VPAC2 (1:50, Abnova, Fisher Scientific, Madrid, Spain) for 1 h at RT. After washing, Alexa Fluor 488 donkey antirabbit IgG and Alexa Fluor 594 goat antimouse IgG (1:500, Invitrogen, Thermo Fisher Scientific) were used as secondary antibodies (1 h at RT). Nuclei staining was performed with 1 mg/mL Hoechst (Sigma-Aldrich). Fluorescence was examined using Leica SP-8 LIGHTNING confocal microscope (Leica DM IRE2; objective, 63×; Leica Microsystems, L’Hospitalet de Llobregat, Spain). Negative controls were performed in the absence of anti-VPAC1 and anti-VPAC2 antibodies. Images were analyzed by FIJI-ImageJ software.

### 2.8. Statistical Analysis and New Variables

Additional variables were defined to elucidate the role of VIP in bone metabolism. We define a coded variable based on the serum concentration of VIP. VIP levels were considered to be low when the concentration was below the 25th percentile of its concentration in the PEARL population (383 pg/mL) and high when the concentration was over the 75th percentile (513 pg/mL).

The data were analyzed using STATA15 (StataCorp LP, College Station, TX, USA) and GraphPad Prism 8.0 software (GraphPad Software, San Diego, CA, USA). Non-parametric variables were analyzed using the Mann–Whitney U test or Kruskal–Wallis and Dunn’s as a *post-hoc* test. *p*-values less than 0.05 were considered significant (* *p* < 0.05; ** *p* < 0.01; *** *p* < 0.001)**.**

## 3. Results

### 3.1. VPAC1 and VPAC2 Receptors Are Expressed by Both Human Monocytes and In Vitro Differentiated Osteoclasts

Since the primary hypothesis underlying our study was the existence of a direct effect of VIP on human osteoclastogenesis and osteoclast function, we decided first to map the expression of VIP receptors in human osteoclast precursors isolated from both healthy donors and early arthritis patients, and in the respective mature osteoclast differentiated in vitro. To determine the expression pattern of VIP receptors in the different cell types, mRNA levels of VIP receptors, VPAC1 and VPAC2, were analyzed by real-time PCR.

**Figure 1:**
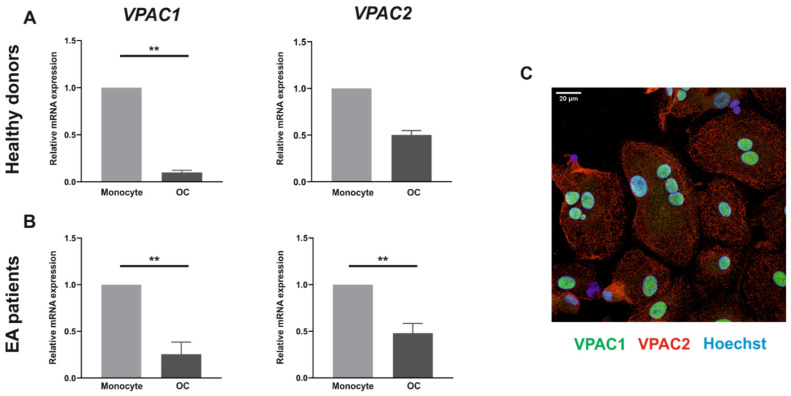
VPAC1 and VPAC2 expression in monocytes and in vitro differentiated osteoclasts. *VPAC1* and *VPAC2* mRNA expression levels in monocytes and in differentiated osteoclasts (OC) from 5 healthy donors (**A**) and 5 early arthritis patients (**B**) was determined by real-time PCR. Results are expressed as relative mRNA expression (relative to *GAPDH* levels). The means ± SEM of triplicate determinations are shown. Mann–Whitney U test was performed (** *p* < 0.01). (**C**) Immunofluorescence analysis on in vitro differentiated osteoclasts from healthy donors using specific antibodies for VPAC1 (Alexa Fluor 488, green) and VPAC2 (Alexa Fluor 594, red). Nuclei were counterstained with Hoechst (blue). Florescence was examined on a Leica SP8 LIGHTNING confocal microscopy.

*VPAC1* and *VPAC2* gene expression was detected in both monocytes and in vitro differentiated osteoclasts. The results showed a generalized reduction in the expression of VIP receptors genes in mature osteoclast. *VPAC1* expression was significantly higher (*p* = 0.041) in monocytes from healthy donors compared with mature osteoclasts (Figure 1A) whereas in early arthritis patients a significant down-regulation of both receptors (*VPAC1* and *VAPC2*) was observed after osteoclastogenesis (*p* = 0.001 and *p* = 0.002, respectively) (Figure 1B).

To confirm the protein expression of VIP receptors in human osteoclasts, immunofluorescence and confocal microscopy detection techniques were performed. The images confirmed the data obtained in the mRNA analysis, showing the presence of both receptors, VPAC1 (green) and VPAC2 (red), in in vitro differentiated osteoclasts (Figure 1C).

All together these results indicate that VPAC1 and VPAC2 are present in both human monocytes and in vitro differentiated osteoclasts showing no differential expression between RA patients and healthy donors.

### 3.2. VIP Limits the In Vitro Osteoclast Differentiation

**Figure 2:**
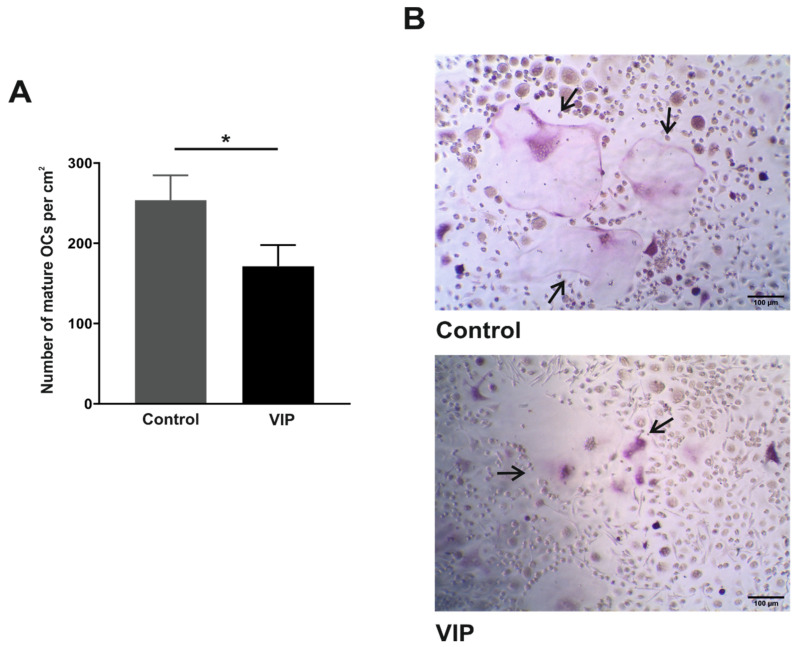
Effect of VIP on osteoclast differentiation. After 14 days of in vitro differentiation osteoclasts were TRAP stained and Hematoxylin was used to visualize the nuclei. TRAP+ cells with 3 or more nuclei were counted. (**A**) Effect of VIP on mature osteoclast number. (**B**) Photomicrographs of the effect of VIP on osteoclast formation. Osteoclasts are indicated with black arrows. The means ± SEM of triplicate determinations of 12 independent experiments are shown. Mann–Whitney U test was performed (* *p* < 0.05).

Once confirmed that VPAC1 and VPAC2 receptors exhibit a similar expression pattern in osteoclasts derived from healthy donors and early arthritis patients, we next elucidated whether VIP was able to affect the differentiation process. To this end, differentiating osteoclasts were exposed to VIP and after 14 days of in vitro culture, mature osteoclasts were quantified considering TRAP-positive multinucleated cells with three or more nuclei. Results revealed a significant decrease in the number of osteoclasts per cm^2^ (*p* = 0.030) when differentiated in the presence of VIP (Figure 2).

### 3.3. Osteoclast Specific Gene Markers Are Negatively Regulated by VIP

Considering the inhibitory action of VIP on osteoclastogenesis reported above, and given that MCSF and RANKL are necessary and enough to induce osteoclast differentiation, we first analyzed whether the presence of VIP during in vitro differentiation modulates the expression of their receptors, cFms and RANK, respectively.

**Figure 3:**
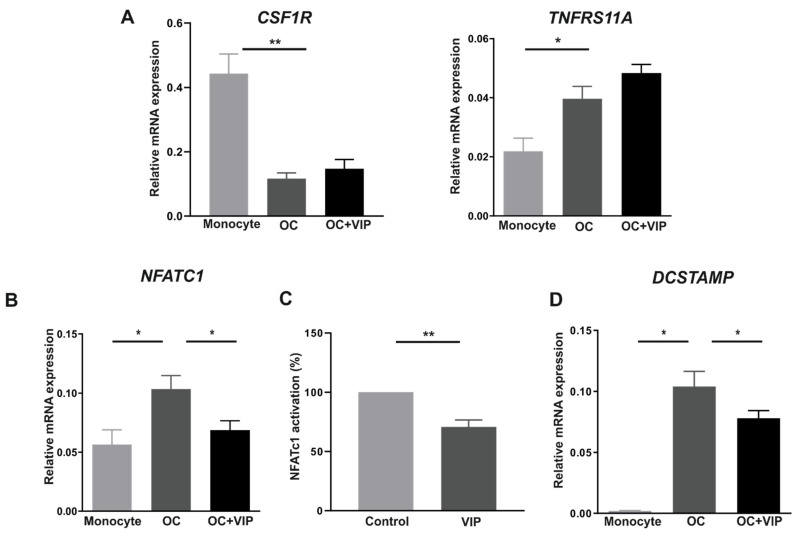
VIP down-regulates the expression of osteoclast-specific markers. *CSF1R*, *TNFRS11A* (**A**) and *NFTAC1* (**B**) mRNA expression levels in monocytes and differentiated osteoclasts in absence (OC) or presence of VIP 10^−8^ M (OC+VIP) was determined by real-time PCR. (**C**) Activation and nuclear translocation of NFATc1 after 12 h of stimulation with 1 ng/mL RANKL (control) or RANKL and VIP 10^−8^ M (VIP). NFATc1 activation was measured in nuclear extracts by TransAM. Results were reported as a percentage increase over control stimulation. (**D**) *DCSTAMP* mRNA expression in monocytes and differentiated osteoclasts in absence (OC) or presence of VIP 10^−8^ M (OC+VIP). Real-time PCR results are expressed as relative mRNA expression (relative to *GAPDH* levels). The means ± SEM of triplicate determinations of 6 independent experiments are shown. Mann–Whitney U test and Kruskal–Wallis using Dunn’s *post hoc* test was performed (* *p* < 0.05; ** *p* < 0.01).

Real-time PCR analyses revealed that the coding gene for cFms (*CSF1R*) was significantly down-regulated after differentiation (*p* < 0.001) whereas the expression of *TNFRS11A*, the gene encoding for RANKL, was significantly up-regulated (*p* = 0.031). No significant effects of VIP in either gene were found (Figure 3A).

To further explore the molecular mechanisms potentially involved in the anti-osteoclastogenic activity of VIP, we then characterized the mRNA expression profiles of *NFATC1* and *DCSTAMP*, two osteoclast specific gene markers, in osteoclasts differentiated in the presence or absence of VIP. Concerning *NFATC1*, results showed a significant decrease in its relative expression in the osteoclasts differentiated in the presence of VIP (*p* = 0.038) (Figure 3B). Given that NFATc1 is considered a master transcription regulator of RANKL-induced differentiation, we next evaluated if the reduction in its expression was also accompanied by a functional change. We studied the activation and nuclear translocation of NFATc1 after 12 h of stimulation with RANKL in presence or absence of VIP. There was a significant decrease of NFTAc1 activation (*p* = 0.002) when the stimulation occurred in presence of the neuropeptide (Figure 3C). Finally, when we studied the expression of *DCSTAMP*, the results revealed that VIP significantly down-regulates *DCSTAMP* expression (*p* = 0.020) in mature osteoclasts (Figure 3D).

### 3.4. Osteoclast Resorptive Activity In Vitro Is Impaired in the Presence of VIP

**Figure 4:**
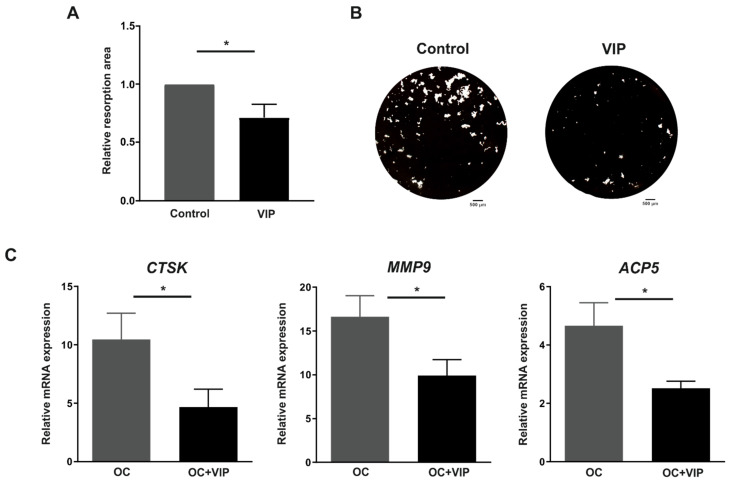
Osteoclast resorptive activity in vitro is decreased by VIP. After 14 days of in vitro differentiation on OsteoAssay^®^ Surface Multiple Well Plate in presence or absence of VIP 10^−8^ M, wells were stained by Von Kossa staining and resorption area was quantified. (**A**) Effect of VIP on resorption area generated by mature osteoclast. Results are presented as percentages relative to the control (mature osteoclasts). (**B**) Photomicrographs of resorption areas formed by osteoclasts differentiated in the absence (control) or presence of VIP (VIP). (**C**) *CTSK*, *MMP9* and *ACP5* mRNA expression levels in differentiated osteoclasts in absence (OC) or presence of VIP 10^−8^ M (OC+VIP) was determined by real-time PCR. Results are expressed as relative mRNA expression (relative to *GAPDH* levels). The means ± SEM of triplicate determinations of 12 independent experiments are shown. Mann–Whitney U test was performed (* *p* < 0.05).

In relation to the possible effect of VIP on the resorbing activity, differentiating osteoclasts grown on OsteoAssay^®^ surface were exposed or not to VIP during the 14 days of culture and the relative resorption area was evaluated. A significant decrease of resorption area was detected when osteoclasts were differentiated in presence of VIP (*p* = 0.014) (Figure 4A,B)

To evaluate the molecular mechanisms mediating the inhibitory effect of VIP on osteoclast resorptive activity, we further analyzed whether the gene expression of molecules involved in bone resorption, including CTSK, MMP9 and TRAP, were modulated in osteoclasts differentiated in the presence of VIP. The results showed that VIP significantly down-regulates the mRNA levels of these three genes: *CTSK*, *MMP9* and *ACP5* (*p* = 0.042, *p* = 0.035 and *p* = 0.022, respectively) (Figure 4C).

### 3.5. The Formation of the Bone Resorption Lacuna Is Interfered by VIP

The formation of the resorption lacuna is dependent on αvβ3 integrin interactions as well as on changes in actin cytoskeleton of osteoclast, thus we next characterized their ex-pression in human osteoclasts differentiated in presence or absence of VIP.

**Figure 5:**
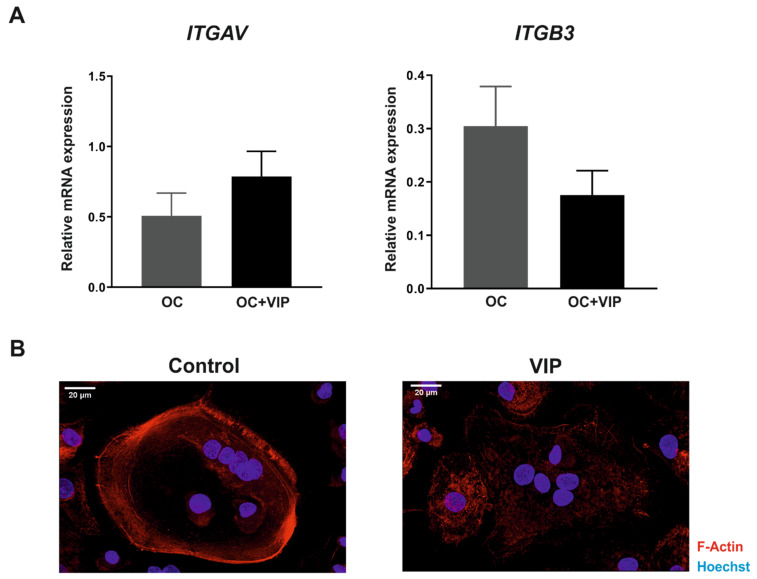
VIP interferes with the formation of the bone resorption lacuna. (**A**) Gene expression of αvβ3 integrin subunits *ITGAV* and *ITGB3* in differentiated osteoclasts in absence (OC) or presence of VIP 10^−8^ M (OC+VIP) was determined by real-time PCR. Results are expressed as relative mRNA expression (relative to *GAPDH* levels). The means ± SEM of triplicate determinations of 6 independent experiments are shown. Mann–Whitney U test was performed. (**B**) Photomicrographs of the effect of VIP on the ring-shaped actin cytoskeleton in differentiating osteoclast. Detection of F-actin was performed using Atto 645N-Phalloidin staining (red), nuclei were counterstained with Hoechst. Florescence was examined on a Leica SP8 LIGHTNING confocal microscopy.

Although no differences in the gene expression of αv subunit (*ITGAV*) were found, β3 subunit (*ITGB3*) transcripts levels tend to decrease when the osteoclastogenesis occurs in presence of VIP (*p* = 0.158) (Figure 5A).

In addition, to elucidate the effect of VIP on the actin ring formation, immunodetection and confocal microscopy techniques were performed. Our results evidenced that VIP treatment during osteoclast differentiation also affects the reorganization of the ring-shaped actin cytoskeleton, where disorganized actin structures were observed (Figure 5B).

### 3.6. VIP Serum Levels Influences Bone Metabolism in Early Arthritis Patients

Next, in light of our in vitro results, we aimed to assess whether there is a relationship between endogenous VIP levels and osteoclast resorption in RA patients. To this end, we evaluated the serum levels of molecules implicated in resorptive activity in early arthritis patients with high and low serum VIP levels.

**Figure 6:**
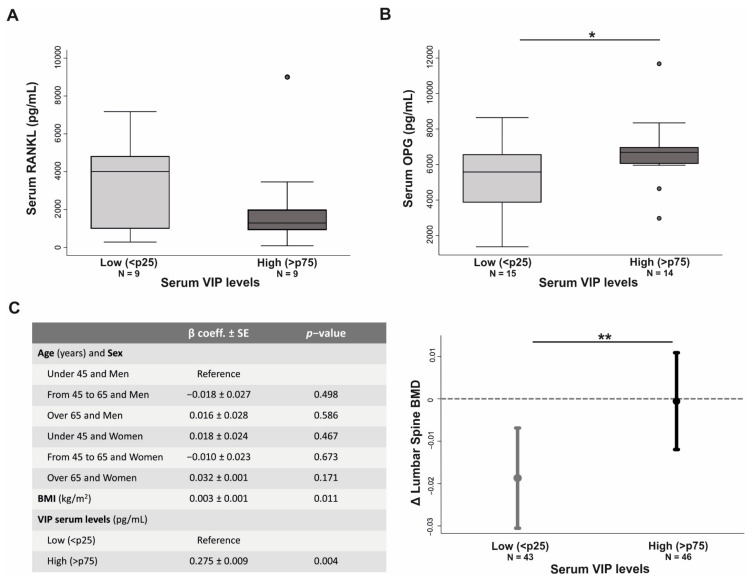
High serum VIP levels reduce bone metabolism markers in patients with early arthritis. Serum concentration of total RANKL (**A**) and OPG (**B**) in early arthritis patients with low (<p25) and high serum (>p75) VIP levels. Data are represented as the interquartile range (p75 upper edge of the box, p25 lower edge, p50 midline), as well as the p90 (line above the box), p10 (line below the box) and dots represents outliers. Mann–Whitney U test was performed. (**C**) Association between serum VIP levels and bone mineral density (BMD) variation from lumbar spine for two years follow-up in early arthritis patients. Data are shown as the mean value of BMD variation for early arthritis patients with low and high serum VIP levels adjusted for the other variables included in the multivariate analysis and the 95% confidence interval. Dotted line indicates no change in lumbar spine BMD (* *p* < 0.05; ** *p* < 0.005).

In addition, we studied whether the serum levels of certain key molecules in bone metabolism could be related to baseline serum VIP levels in patients with early arthritis. Results revealed a trend towards a decrease in serum RANKL levels (*p* = 0.27) as well as a significant increase in OPG concentration in those patients with higher serum VIP levels (*p* = 0.04) (Figure 6A,B).

Finally, to further explore the potential connection between VIP and bone integrity in patients with RA, we studied the effect of serum VIP levels on bone mineral density (BMD) variation after two years follow-up in RA patients. In view of theconfounding factors that could influence BMD variation, we performed a multivariate regression model with the next criteria: BMD variation as dependent variable, Body Mass Index (BMI) and the intersection between age of disease onset and sex as independent confounding factors, and VIP serum levels as independent variable. After adjustment for these confounders, our statistical analysis showed that having high serum VIP concentration was significantly associated (*p* = 0.004) with a lower BMD lost (Figure 6C).

## 4. Discussion

The imbalance between bone resorption and formation results in an excessive destruction of bone in rheumatic joints. In fact, an increased osteoclast differentiation and resorptive activity is recognized as a characteristic feature of RA [1,40,41]. Osteoclastogenesis is a multistep process controlled by complex neuro-immune-osteogenic interactions mediated by systemic hormones, local cytokines and neuropeptides. Among them, VIP has been proven to act as a negative regulator of differentiation and resorptive activity of rat and mouse osteoclasts [28,29,30,38], demonstrating a protective action on bone destruction in experimentally-induced arthritis [36,37]. However, the role of VIP in human osteoclastogenesis remains unexplored. To our knowledge, here we investigated for the first time the effect of VIP in human osteoclast formation and activity, corroborating its inhibitory action and unraveling the underlying molecular mechanisms and signaling pathways involved. Furthermore, we found a connection between serum levels of VIP and key regulators of osteoclastogenesis in early arthritis patients that corresponds with its bone-protective effects described in the CIA model [36,37], suggesting that VIP may contribute to improving bone homeostasis in these patients.

Our findings evidence the presence of VPAC1 and VPAC2 receptors in human osteoclast precursors and mature osteoclast differentiated in vitro, in accordance with those previously described in mouse and rat bone marrow-derived osteoclasts [28,38,42,43]. We also found that VIP receptor transcripts levels were reduced at the end of the differentiation process, showing an expression pattern consistent with that recently described in the differentiation of mouse pre-osteoclasts from bone marrow-derived macrophages [38]. These results are also in accordance with the lesser expression of VIP receptors in the human monocyte to macrophage differentiation in vitro [44], suggesting that the myeloid cell differentiation process would entail a reduction in the expression of VPAC1 and VPAC2. However, further studies are needed to determine the functional implications of these changes in the expression pattern of VIP receptors.

When we examined the modulatory action of VIP on the in vitro differentiation of human osteoclasts, a significant decrease in the number of mature osteoclasts formed was observed, in agreement with that previously reported in studies on murine models [29,38]. The molecular mechanism potentially involved in this inhibitory action showed that VIP does not affect the gene expression of receptors for M-CSF and RANKL, two critical cytokines for inducing the differentiation process [8,45]. Given that the expression of RANKL receptor is dependent on signals induced by M-CSF [46], not finding a VIP effect on the cell-surface receptor c-Fms could explain the lack of change in transcripts for RANK. Conversely, in osteoclasts formed in the presence of VIP, we detected a decrease in the expression levels of *NFTAC1* and *DCSTAMP* genes, identified as markers of osteoclast differentiation [8]. In this regard, the VIP-induced reduction of NFATc1 transcription factor is particularly noteworthy given that it is recognized as the master regulator of osteoclast formation and function [47]. As NFκB and c-Fos are upstream of NFATc1 [48], and VIP has been shown to prevent the NFκB and AP-1 nuclear translocation [49], this might be involved in the decreased expression of NFATc1 reported. Indeed, Qu et al. have recently reported that VIP treatment suppresses the NFκB signaling pathway in RANKL-induced rat osteoclast formation [30]. Moreover, our findings proved that VIP also inhibits the RANKL-induced activation and nuclear translocation of NFATc1, consistent with the reduced transcriptional activity of NFAT isoform c3 (NFATc3) described in primary pulmonary artery smooth muscle cells after VIP treatment [50]. Indeed, we further reported the down-regulation of the NFATc1-regulated *DCSTAMP* gene in the presence of VIP. DC-STAMP is a protein critical for mononuclear osteoclast fusion [51], therefore a reduction in its expression could validate the decrease of mature osteoclast number observed when precursors were exposed to VIP. Taken together, our results suggest that VIP, rather than affecting the early steps of MCSF/RANKL-mediated osteoclastogenesis, exerts its modulatory action downstream of this signaling pathway by negatively regulating NFATc1 expression and activation.

This promising anti-osteoclastogenic potential of VIP was reinforced by functional studies of in vitro resorption assay. Osteoclasts generated in the presence of VIP displayed a reduced matrix resorption activity, in accordance with previous findings in rat osteoclasts [28,30,52]. In addition, we found a decrease in the mRNA levels of proteolytic enzymes involved in osteoclast resorption, including CTSK, MMP9, and TRAP, three mediators regulated by NFATc1 [53,54,55,56]. A protease-signaling network has been suggested to explain their regulation. MMP9 is produced as a latent pro-enzyme, thus requiring removal of a prodomain which can be elicited by CTSK in the resorption lacuna. In turn, CTSK is endocytosed along with bone breakdown products into the osteoclast to finally fuse with TRAP-containing vesicles. This allows TRAP to be cleaved and activated by CTSK in the transcytotic vesicles where it will complete the degradation of organic components of bone [57,58]. Thus, the lesser expression of these three enzymes observed in the presence of VIP is likely to have an impact on the resorptive activity of osteoclasts which could be also explained by the negative modulation of NFATc1. Additionally, and in agreement with our results, it had already been described that VIP-signaling induces a reduction in MMP9 expression in rodent models of colitis and lung damage [59,60] as well as in fibroblastic synoviocytes from patients with osteoarthritis [61].

Adhesion of osteoclasts to the bone matrix and cell migration, both processes mediated by the membrane integrin αvβ3, are essential initial steps for bone resorption [7,62]. Subsequently, reorganization of the actin cytoskeleton into a peripheral ring that also involves integrins takes place in podosomes, forming a sealing zone which isolates the resorption lacuna from the environment [6,63]. Interestingly, our results showed that osteoclast differentiation in the presence of VIP influences integrin expression as well as actin ring formation. We detected a clear trend in the reduction of integrin β3 subunit mRNA level, which is concordant with our findings since the *ITGB3* gene is a direct target of the VIP-negatively regulated NFATc1 transcription factor [64]. Although there are no previous data regarding the impact of VIP on actin ring formation, Lundberg et al. reported that VIP reduces the number of pits in rat osteoclast cultures by inducing a rapid cytoplasmic contraction and reduced motility, both effects attributable to an altered actin cytoskeleton reorganization [28]. Therefore, results related to osteoclast activity suggest that VIP impairs bone resorption by down-regulating proteolytic enzymes and by disrupting two vital molecular mechanisms underlying the creation of a functional resorption lacuna, i.e., αvβ3 integrin-dependent interactions and changes in actin filament organization.

Given that osteoclasts derived from early arthritis patients express VPAC receptors and VIP opposes the osteoclastogenic effects of M-CSF and RANKL and reduces bone resorption in vitro, we hypothesized that the VIP/VPAC receptors axis, through the action of endogenous VIP, may be involved in bone function. As a first approach to test our translational hypothesis, and considering that VIP-induced decrease of the RANKL/OPG has been involved in its protective effect on bone destruction in the CIA model [36], we analyzed their serum levels in early arthritis patients clustered according to VIP serum levels. Patients with high VIP levels showed a lower concentration of RANKL which, although not statistically significant, is noteworthy as significantly increased levels of OPG were also found, thus resulting in a decrease in the RANKL/OPG ratio. Interestingly, an analogous reduction of the RANKL/OPG balance associated with a decrease in osteoclast activity has been shown to be induced by VIP in vitamin D-stimulated osteoclastogenesis in mouse marrow cultures [29] as well as in rat osteoclasts [30]. Moreover, we further found that early arthritis patients with elevated serum VIP levels exhibit lower BMD loss, a clinical marker for bone strength that has been associated with joint damage in RA [19,21,22]. Consequently, our data would imply that VIP might dampen the osteoclastic component in RA pathogenesis by modulating the RANKL/OPG ratio in favor of OPG. These findings would agree with recent studies showing that serum VIP levels can be used as a prognostic biomarker in early arthritis given that, although no differences in serum neuropeptide levels between patients and healthy donors have been reported, those patients with low baseline levels have a poorer clinical course [34,35].

In summary, our results from the differentiation of human osteoclasts in vitro in the presence of VIP indicate that its direct anti-osteoclastogenic effect is present in human cells, impairing both differentiation and resorption activity, mainly through the negative regulation of NFATc1. In addition, our findings on the relationship between serum levels of VIP and bone metabolism markers in early arthritis patients are in line with our hypothesis about bone-protective effects of VIP in humans, and also point out that in addition to exerting a direct inhibitory effect on osteoclasts, this neuropeptide seems to regulate the RANK/RANKL/OPG system. Therefore, although other molecular mechanisms underlying the VIP anti-osteoclastogenic effects need to be explored, our present results could contribute to establishing the basis to validate the potential of VIP in the improvement of bone homeostasis in RA patients.

## Data Availability

The data presented in this study are available on request from the corresponding author. The data are not publicly available due to the lack of an existing repository.
